# Quantum computing in finance: a literature review and future directions for trustworthy financial AI

**DOI:** 10.3389/frai.2026.1929828

**Published:** 2026-08-27

**Authors:** Silvia Muzzioli, Farhana Raheem, Massimiliano Ferrara, Paolo Giudici, Roberta Siciliano

**Affiliations:** 1 Marco Biagi Department of Economics, University of Modena and Reggio Emilia, Modena, Italy; 2 Department of Law, Economics and Human Sciences, Mediterranea University of Reggio Calabria, Reggio Calabria, Italy; 3 Department of Economics and Management, University of Pavia, Pavia, Italy; 4 Department of Electrical Engineering and Information Technologies, University of Naples Federico II, Naples, Italy

**Keywords:** applied finance, fuzzy logic, portfolio optimization, quantum computing, safe AI

## Abstract

The paper provides an integrated literature review of recent scientific publications on quantum computing in finance and identifies promising directions for future research on the subject. The review covers seven thematic areas: portfolio optimization, derivative pricing and stochastic volatility, quantum machine learning for fraud detection and credit risk, insurance and actuarial science, mixed-frequency econometrics, fuzzy-quantum approaches for financial explainability, and security of cryptocurrencies. The paper compiles the essential quantum computational methods proposed in the literature, outlines their economic significance and the existing constraints for empirical testing and implementation, and discusses cross-cutting issues of explainability, trustworthy AI, robustness, and governance that arise across these application domains. Drawing on this review, the paper identifies five macro-gaps in the existing literature and proposes seven concrete directions for future research, grounded in European financial data and currently available quantum computing infrastructure. A special focus throughout is the increasingly available quantum infrastructure in Europe and the regulatory emphasis on trustworthy artificial intelligence, both of which create timely opportunities for future applications in financial modelling, risk management, and explainable financial AI.

## 1 Introduction

Financial decision-making increasingly relies on solving computationally intensive problems involving high-dimensional optimization, stochastic simulation, risk forecasting, and machine learning. Portfolio construction, derivative pricing, credit risk assessment, fraud detection, and insurance modelling all require analysing large and complex datasets while accounting for multiple sources of uncertainty. Although classical computing has enabled remarkable advances in these areas, many financial problems remain computationally demanding because of their combinatorial complexity and the increasing volume of available information. In this context, quantum computing has emerged as a promising computational paradigm capable of exploiting superposition, interference, and entanglement to tackle specific classes of optimization, simulation, and learning problems more efficiently than classical algorithms.

The application of quantum computing to finance has evolved rapidly over the last few years. Early research mainly focused on theoretical developments, demonstrating how quantum algorithms could be adapted to classical financial problems such as portfolio optimization, derivative pricing, and risk analysis (Egger et al., 2020). This body of work builds on a broader physics finance crossover, in which concepts and methods from statistical physics and quantum mechanics have long informed the modelling of financial markets, providing part of the conceptual foundation for the later development of quantum financial algorithms. More recently, advances in noisy intermediate-scale quantum (NISQ) hardware and hybrid quantum-classical algorithms have stimulated a growing body of research investigating practical applications in portfolio management, stochastic volatility estimation, fraud detection, credit scoring, insurance, and cryptocurrency security (Herman et al., 2023; Naik et al., 2025). Although current quantum hardware is still limited by noise and scalability constraints, the continuous development of commercial quantum platforms and public research infrastructures has transformed quantum finance from a largely theoretical discipline into an active and rapidly expanding research area.

Several recent surveys have reviewed the emerging literature on quantum computing in finance (Egger et al., 2020; Herman et al., 2023; Naik et al., 2025). Among these, Herman et al. (2023) provide a particularly comprehensive baseline for mapping classical financial problems onto quantum circuits across optimization, simulation, and machine learning; the present review differs by concentrating on European financial data, EU regulatory frameworks such as the EU AI Act, and an explicit trustworthy-AI and governance agenda rather than on algorithmic taxonomy alone. It should be noted that much of the evidence surveyed in these works concerns theoretical speed-ups rather than empirically observed advantage on hardware. Rather than extending the existing algorithm-oriented surveys, the present review complements them by placing empirical validation, trustworthy AI, governance, and the European research ecosystem at the centre of the discussion. More broadly, Rohan et al. (2026) provide a comprehensive review of artificial intelligence and machine learning methods for stock market prediction, including a discussion of quantum computing as one of several emerging technologies with potential financial applications. However, the existing reviews primarily focus on quantum algorithms, computational complexity, or hardware developments, and treat quantum computing either as one component among many AI techniques or without dedicated attention to European financial markets, the governance and explainability requirements of the EU AI Act, or the practical research agenda enabled by European quantum computing infrastructure. Comparatively less attention has been devoted to providing an integrated overview organised around financial applications, identifying common methodological challenges across different domains, and discussing the opportunities created by the rapidly evolving European quantum ecosystem. Furthermore, much of the existing literature remains based on proof-of-concept studies using relatively small datasets or simplified financial settings, while empirical validation on real-world financial data is still limited.

This paper aims to provide a comprehensive review of the current literature on quantum computing in finance while identifying promising directions for future research. Rather than focusing exclusively on computational aspects, the review adopts an application-oriented perspective and organises the literature into seven major domains: portfolio optimization; derivative pricing and stochastic volatility; quantum machine learning for fraud detection and credit risk; insurance and actuarial science; mixed-frequency econometrics; fuzzy-quantum approaches for financial decision-making; and cryptocurrency security. For each area, we discuss the main quantum computational approaches, the current state of empirical evidence, and the principal limitations that remain to be addressed.

A distinctive feature of this review is its emphasis on the European research landscape. Recent investments in quantum computing infrastructure, including the resources made available through CINECA and the EuroHPC initiative, provide new opportunities for conducting empirical research on quantum financial applications. At the same time, the European regulatory environment places increasing emphasis on trustworthy artificial intelligence, transparency, and responsible innovation, creating a particularly interesting setting for future developments at the intersection of quantum computing, artificial intelligence, and finance.

The paper makes four distinctive contributions to the emerging literature on quantum finance. First, it provides an up-to-date and application-oriented synthesis of the rapidly expanding literature across seven financial domains. Second, it identifies common methodological challenges and research gaps that cut across these application areas. Third, it develops a European research agenda by linking quantum finance to European datasets, quantum infrastructures, and regulatory frameworks such as the EU AI Act, the Markets in Crypto-Assets Regulation (MiCA), and Solvency II. Fourth, it proposes a framework for trustworthy financial AI by integrating quantum computing with explainability, governance, and Security, Accuracy, Fairness and Explainability (SAFE AI) principles.

The remainder of the paper is organised as follows. Section 2 describes the review methodology, surveys the literature on quantum computing in finance across seven thematic areas, discusses cross-cutting issues of explainability, trustworthy AI, robustness, and governance, and identifies the main research gaps emerging from this review. Section 3 proposes seven directions for future research building on these gaps. Section 4 concludes.

## 2 Review methodology

This study adopts a structured narrative review methodology aimed at providing a comprehensive overview of the rapidly evolving literature on quantum computing in finance. Given the interdisciplinary nature of the field, which combines quantum computing, artificial intelligence, financial engineering, operations research, and computational economics, the objective of the review is not to perform a statistical meta-analysis but rather to synthesise the existing body of knowledge, identify the principal research streams, highlight common methodological challenges, and discuss promising directions for future research.

The literature search was conducted using major academic databases and scientific repositories, including Google Scholar, Scopus, Web of Science, IEEE Xplore, SSRN, and arXiv. Additional institutional reports and technical publications from leading organisations active in quantum computing, including IBM Quantum, D-Wave Systems, IQM, JPMorgan Chase, the World Economic Forum, EuroHPC, and CINECA, were also considered when relevant to the development of practical financial applications.

The search strategy combined keywords related to both quantum computing and financial applications. Representative search terms included quantum finance, quantum portfolio optimization, quantum machine learning, quantum derivative pricing, quantum stochastic volatility, quantum risk management, quantum actuarial science, quantum MIDAS (Mixed Data Sampling), quantum blockchain, quantum cryptocurrency, fuzzy quantum systems, and financial quantum computing. Reference lists of the selected publications were also examined to identify additional relevant studies.

The review primarily covers publications appearing between 2018 and 2026, a period corresponding to the rapid expansion of research on hybrid quantum-classical algorithms and noisy intermediate-scale quantum (NISQ) computing. Earlier contributions are included when they provide the theoretical foundations of the field, such as seminal works on portfolio theory, option pricing, fuzzy systems, quantum algorithms, and mixed-frequency econometrics.

Rather than organising the literature according to quantum algorithms alone, this review adopts an application-oriented classification framework, reflecting the perspective of financial researchers and practitioners. The reviewed studies are grouped into seven thematic areas: (i) portfolio optimisation; (ii) derivative pricing and stochastic volatility; (iii) quantum machine learning for fraud detection and credit risk; (iv) insurance and actuarial science; (v) quantum econometrics and mixed-frequency modelling; (vi) fuzzy-quantum approaches for financial decision-making; and (vii) cryptocurrency security and post-quantum cryptography.

Within each thematic area, the literature is analysed according to five complementary dimensions: the financial problem addressed, the quantum computational approach employed, the level of empirical validation, the quantum hardware or software platform considered, and the principal research limitations identified by the existing literature. This multidimensional perspective allows common methodological challenges to be identified across different application domains while highlighting opportunities for future interdisciplinary research. Within each thematic area, studies were designated as key references in Table 1 on the basis of three criteria: being among the first to propose a given quantum approach, providing comparatively comprehensive empirical or hardware validation, or serving as a widely cited foundational reference for the method.

**Table 1:** Taxonomy of quantum finance literature. Summary of the principal application domains reviewed, representative quantum approaches, hardware platforms, and key references.

Application area	Main financial task	Representative quantum approaches	Typical hardware platform	Key references
Portfolio Optimization	Asset allocation and portfolio construction	QAOA, VQE, Quantum Annealing	IBM Quantum, D-Wave	Herman et al. (2023); Buonaiuto et al. (2023); Brandhofer et al. (2023)
Derivative Pricing and Stochastic Volatility	Option pricing, risk analysis, volatility estimation	Quantum Amplitude Estimation (QAE), Quantum Hidden Markov Models (QHMM), Quantum Monte Carlo	IBM Quantum, quantum simulators	Woerner and Egger (2019); Ghysels et al. (2023, 2025); Zhou (2025)
Quantum Machine Learning	Fraud detection, credit scoring, financial classification	Variational Quantum Classifiers (VQC), Quantum Kernels, QBoost	IBM Quantum, D-Wave	Doosti et al. (2026); Rath and Date (2025); Sasikala and Sivakami (2025)
Insurance and Actuarial Science	Loss simulation, mortality modelling, reinsurance optimisation	Quantum Amplitude Estimation, Quantum Annealing	IBM Quantum, quantum simulators	Liu et al. (2025); Tamturk and Cortis (2023)
Quantum Econometrics	Mixed-frequency forecasting, MIDAS models	Quantum linear systems algorithms	Quantum simulators	Berkowitz (2025); Ghysels et al. (2004)
Fuzzy–Quantum Systems	Explainable financial decision-making, rule optimisation	Quantum Annealing, Quantum Fuzzy Inference	D-Wave	Pourabdollah et al. (2022); Acampora et al. (2023)
Cryptocurrency and Blockchain	Post-quantum cryptography, blockchain security	Shor’s algorithm, Grover’s algorithm	Future fault-tolerant quantum computers	Almuhammadi and Alghamdi (2025); Babbush et al. (2026); Bonaparte (2025)

QAOA, Quantum Approximate Optimization Algorithm; VQE, Variational Quantum Eigensolver; VQA, variational quantum algorithm; QAE, Quantum Amplitude Estimation; QHMM, Quantum Hidden Markov Model; QUBO, Quadratic Unconstrained Binary Optimization; QNN, quantum neural network; FFNN, feedforward neural network; DNN, deep neural network; GBM, geometric Brownian motion; HHL, Harrow-Hassidim-Lloyd.

The purpose of the review is therefore not only to summarise the current state of knowledge but also to provide a coherent conceptual framework for understanding the evolution of quantum finance as an emerging research field at the intersection of quantum computing, artificial intelligence, and financial decision-making. The principal application domains reviewed, together with their representative quantum approaches, typical hardware platforms, and key references, are summarised in Table 1.

### 2.1 Quantum portfolio optimization

Portfolio optimization is widely regarded as one of the most promising applications of quantum computing in finance. Since the seminal work of Markowitz (1952), portfolio construction has evolved from a quadratic optimisation problem into a large-scale combinatorial optimisation problem once practical constraints such as transaction costs, cardinality limits, ESG (environmental, social and governance) requirements and regulatory capital constraints are introduced. Quantum approaches to portfolio optimization can be broadly classified into two main algorithmic families. One algorithmic family is variational quantum algorithms like QAOA (Quantum Approximate Optimization Algorithm, Farhi et al., 2014; Farhi and Harrow, 2016) and VQE (Variational Quantum Eigensolver). In both cases, the portfolio question is reexpressed as a QUBO (Quadratic Unconstrained Binary Optimization) model, where the decision to hold, or not hold, an asset becomes a binary variable and both the objective function and the portfolio constraints are then incorporated into a cost Hamiltonian. After that, a parameterized quantum circuit prepares a quantum state, and then a classical optimizer keeps adjusting the circuit parameters, over and over, to drive down the expected cost. This mixed quantum-classical cycle is meant to work on today’s NISQ devices, without asking for fault-tolerant quantum computers. In fact, Buonaiuto et al. (2023) showed practical best practices for this kind of workflow using real IBM hardware. Brandhofer et al. (2023) provided rigorous benchmarking, concluding that at current qubit counts the method functions as a heuristic competitive with classical heuristics on small instances but performance improves systematically with circuit depth.

The second algorithmic family consists of quantum annealing on D-Wave hardware, where the portfolio QUBO is embedded directly into the annealer’s native topology without circuit compilation. D-Wave Advantage with more than 5,000 qubits in the Pegasus topology is the most immediately practical quantum platform for portfolio optimization and is accessible through CINECA’s ISCRA programme. More recently, D-Wave has moved toward a dual-platform strategy: while continuing to develop its annealing systems, the company announced in 2026 an accelerated gate-model roadmap, targeting an initial gate-based system supported by its acquisition of Quantum Circuits, Inc. (D-Wave, 2026). This convergence suggests that the historical separation between annealing and gate-based approaches may narrow in the coming years. IBM and Vanguard (2024) jointly demonstrated a sampling-based variational quantum algorithm for bond ETF construction. JPMorgan Chase (2024) introduced Hybrid HHL++ (Harrow-Hassidim-Lloyd), tested on Quantinuum H-series hardware, representing the largest hardware demonstration of the HHL linear systems algorithm for a financial portfolio problem to date.

Overall, portfolio optimisation represents the most mature application of quantum computing in finance, both in terms of algorithmic development and experimental validation. The existing literature has significant limitations. The great majority of results are proof-of-concept demonstrations on N < 50 assets with idealized constraint sets. No existing study benchmarks quantum portfolio results against CPLEX and Gurobi the industry-standard classical optimization solvers on problem instances that include the full simultaneous set of cardinality constraints, ESG mandates, Basel IV risk-weighted asset floors, transaction cost penalties, and liquidity buffers. While a recent study demonstrates quantum-informed portfolio selection on European equity indices such as the DAX and FTSE 100 using trapped-ion hardware (Yalovetzky et al., 2026), no existing study integrates the full simultaneous set of European ESG mandates, liquidity, transaction-cost and prudential constraints. Whether quantum or hybrid methods provide any measurable advantage over best-in-class classical alternatives on such realistic European instances remains an open empirical question. Despite significant progress, current evidence remains largely confined to proof-of-concept studies involving relatively small asset universes and simplified portfolio constraints (Herman et al., 2023). Consequently, it is still unclear whether quantum algorithms provide a measurable advantage over state-of-the-art classical optimisation methods in realistic institutional portfolio management problems. Addressing this question remains one of the most important research challenges in quantum finance (Tables 2–6).

**Table 2:** Representative quantum portfolio optimization studies and implementations.

Study	Quantum approach	Hardware/platform	Problem/dataset	Classical baseline	Validation
Buonaiuto et al. (2023)	Variational (QAOA/VQE), QUBO	Real IBM hardware	Asset allocation, small N	-	Real-device workflow
Brandhofer et al. (2023)	QAOA	IBM + simulator	Small instances	Classical heuristics	Competitive on small N
IBM and Vanguard (2024)	Sampling-based VQA	IBM Quantum	Bond ETF construction	-	Applied demonstration
JPMorgan Chase (2024)	Hybrid HHL++	Quantinuum H-series	Portfolio (linear systems)	Noiseless simulation/analytical solution	Largest HW demo to date
D-Wave Advantage	Quantum annealing	D-Wave (Pegasus, 5,000 + q)	QUBO with cardinality	-	Native-topology embedding

**Table 3:** Representative quantum derivative pricing and stochastic volatility studies.

Study	Quantum approach	Hardware/platform	Problem/dataset	Classical baseline	Validation
Woerner and Egger (2019)	Quantum Amplitude Estimation	IBM Quantum	VaR/ES risk analysis	Classical Monte Carlo	Proof-of-concept
Stamatopoulos et al. (2020)	QAE option pricing	IBM Quantum	European option, single-asset GBM	Monte Carlo	Proof-of-concept
Ghysels et al. (2023)	Quantum algorithms, regime-switching	Simulator	Derivative pricing + credit risk	Classical regime-switching	Theoretical
Ghysels and Morgan (2024)	Quantum states, dynamic asset pricing	Simulator	Nonlinear dynamic asset pricing	Classical solvers	Theoretical
Ghysels et al. (2025)	Quantum Hidden Markov Models	Simulator	Stochastic volatility filtering	Heston, GARCH, HMM	Theoretical bounds
Zhou (2025)	Quantum Monte Carlo (overview)	-	Derivative pricing, risk	Classical MC	Survey

**Table 4:** Representative quantum machine learning studies in finance.

Study	Quantum approach	Hardware/platform	Problem/dataset	Classical baseline	Validation
Doosti et al. (2026)	QML survey	—	Fraud, credit, trading	Classical ML	Survey
Chen et al. (2025)	Hybrid QNN, amplitude encoding	Simulator	Credit recovery rate (regression)	FFNN, angle-enc., XGBoost	Empirical
Bakshi and Srinivasan (2025)	Qubit–qutrit neural network	Simulator	Real-time forecasting (regression)	Classical NN	Empirical
Sasikala and Sivakami (2025)	QNN + Quantum Autoencoder	Simulator	Fraud/risk, small data	Classical NN	Empirical (small data)
Rath and Date (2025)	Hybrid quantum-classical DNN	Simulator	UCI German Credit	XGBoost, classical NN	Empirical
Intesa Sanpaolo–IBM (World Economic Forum, 2025)	Variational quantum classifier	IBM Quantum	Payment fraud	Classical classifiers	Deployment report

**Table 5:** Representative quantum insurance and actuarial studies.

Study	Quantum approach	Hardware/platform	Problem/dataset	Classical baseline	Validation
Liu et al. (2025)	Quantum stochastic modelling; optimization	Simulator	Non-life/life/reinsurance	Classical actuarial	First systematic study
Tamturk and Cortis (2023)	Compound Poisson simulation	IBM Qiskit simulator	Aggregate loss processes	Classical simulation	Feasibility

**Table 6:** Mapping of the five macro-gaps (Section 2.9) to the seven proposed research directions.

Macro-gap (Section 2.9)	Addressed by research direction(s)
Gap 1 Quantum econometrics (QHMM, MIDAS)	3.1 (QHMM, European markets); 3.2 (Quantum MIDAS)
Gap 2 Portfolio optimization under full European constraints	3.3 (Full-constraint European portfolio)
Gap 3 Cross-sector anomaly, default and risk detection	3.4 (Cross-sector European anomaly, default and risk detection)
Gap 4 Actuarial science under Solvency II	3.5 (European actuarial science)
Gap 5 Explainability, robustness, governance	3.6 (Fuzzy-quantum explainability); 3.7 (Robustness & governance)

### 2.2 Quantum derivative pricing and stochastic volatility

Derivative pricing and stochastic volatility modelling are among the most computationally demanding problems in quantitative finance. Valuing financial derivatives typically requires estimating expectations over complex stochastic processes, while volatility modelling involves inferring latent state variables from noisy market observations. Classical Monte Carlo simulation remains the standard numerical approach for many pricing problems, but its computational cost increases rapidly with model complexity and the number of simulated scenarios. Consequently, these problems have attracted considerable attention within the quantum finance literature.

One of the earliest and most influential quantum approaches to derivative pricing is Quantum Amplitude Estimation (QAE) (Brassard et al., 2002), which theoretically provides a quadratic speed-up over classical Monte Carlo methods by improving the convergence rate from O(1/√N) to O(1/N). Building on this framework, Woerner and Egger (2019) demonstrated the application of QAE to financial risk analysis, including the estimation of Value-at-Risk (VaR) and Expected Shortfall (ES). Subsequently, Stamatopoulos et al. (2020) implemented quantum algorithms for European option pricing on IBM quantum hardware. Although these studies provided important proof-of-concept demonstrations, their empirical applications were restricted to relatively simple single-asset geometric Brownian motion models and did not incorporate stochastic volatility or regime-switching dynamics.

More recent research has extended quantum methods beyond option pricing toward dynamic asset pricing and volatility estimation. A major contribution in this direction is the research programme developed by Ghysels and collaborators. Ghysels et al. (2023) proposed quantum algorithms for derivative pricing and credit risk within a regime-switching economy, allowing option valuation and credit risk assessment to be performed simultaneously within a unified quantum framework. Ghysels and Morgan (2024) further demonstrated that equilibrium solutions to nonlinear dynamic asset pricing models can be represented as quantum states, showing the potential for substantial computational gains in solving high-dimensional dynamic financial models.

An important recent development concerns the estimation of stochastic volatility. Ghysels et al. (2025) introduced Quantum Hidden Markov Models (QHMMs) for maximum-likelihood estimation and filtering of latent volatility processes. In contrast with classical stochastic volatility models such as Heston (1993), GARCH, or conventional Hidden Markov Models, the proposed framework exploits quantum state representations and quantum amplitude amplification to accelerate likelihood evaluation and filtering. Their theoretical analysis suggests tighter non-asymptotic estimation bounds than those achieved by classical methods, indicating that quantum algorithms may improve both computational efficiency and statistical precision in volatility estimation. This represents a concrete and measurable improvement over classical volatility estimation methods (Mancino and Recchioni, 2015). Zhou (2025) provides a recent overview of quantum Monte Carlo methods and their implications for derivative pricing and financial risk measurement.

Despite these important theoretical advances, empirical validation remains limited. Most existing studies have been evaluated on simulated data or highly simplified financial settings, while applications to real financial markets remain scarce. In particular, European option markets have received very little attention despite offering a rich source of forward-looking information on market expectations. Model-free implied variance, implied skewness and implied kurtosis, constructed following Bakshi et al. (2003), together with the variance risk premium (Carr and Wu, 2009), provide valuable information on future volatility dynamics and tail risk that has not yet been incorporated into existing quantum stochastic volatility models. Similarly, recently developed risk-asymmetry measures based on European option markets (Elyasiani et al., 2025) offer additional information on volatility regimes that could complement quantum state estimation.

Overall, derivative pricing and stochastic volatility estimation represent one of the most theoretically advanced areas of quantum finance. However, the transition from algorithmic developments to large-scale empirical applications remains at an early stage. Future research should therefore focus on validating quantum pricing and volatility models using real financial datasets, benchmarking them against state-of-the-art classical approaches, and assessing their practical performance under realistic hardware constraints.

### 2.3 Quantum machine learning in finance

Among the various applications of quantum computing in finance, quantum machine learning has experienced the fastest growth during the last five years (Doosti et al., 2026), driven by the increasing adoption of AI-based decision systems in banking, insurance and financial services. Quantum Machine Learning (QML) refers to the use of quantum computing for classification, regression, and anomaly detection tasks. Its theoretical potential lies in quantum kernel methods, which compute inner products in an exponentially large feature space, and in variational quantum circuits (VQCs), which function as quantum neural networks trained within hybrid quantum-classical architectures.

Doosti et al. (2026) surveyed QML applications for financial services, finding that fraud detection, credit scoring, and algorithmic trading offer the most immediate near-term potential, although advantages over classical methods on real financial datasets remain limited (Fritz-Morgenthal et al., 2022). Rohan et al. (2026) similarly identify quantum machine learning as a promising direction for stock price prediction, noting the potential of quantum-enhanced portfolio optimization and quantum-inspired classifiers, while acknowledging that current NISQ hardware limitations restrict practical deployment. The empirical evidence to date suggests that quantum computing currently shows greater promise for classification problems, involving discrete outcomes such as credit approval or fraud detection, than for regression problems, which require predicting continuous values such as prices or volatility. This is because the financial applications with the strongest empirical evidence of quantum advantage are those involving binary or categorical response variables (Naik et al., 2025). Recent evidence complicates this picture, however: Chen et al. (2025) demonstrated that a hybrid quantum neural network using Amplitude Encoding outperformed both classical feedforward neural networks and quantum models using Angle Encoding in recovery rate prediction, a continuous regression task, and performed competitively with XGBoost, suggesting that the classification advantage of quantum methods may not be universal. Reinforcing this point, Bakshi and Srinivasan (2025) show that quantum-inspired qubit-qutrit neural networks achieve competitive real-time financial forecasting on continuous time-series data, providing further evidence that hybrid quantum architectures are beginning to advance regression and forecasting tasks previously considered less amenable to quantum methods. These results are, however, obtained on quantum simulators and do not yet demonstrate an advantage on real quantum hardware.

Several studies have proposed quantum approaches specifically for fraud detection and credit risk. Sasikala and Sivakami (2025) developed a hybrid framework combining Quantum Neural Networks with Quantum Autoencoders, which outperformed classical neural network baselines on smaller datasets and with fewer trainable parameters. In a practical deployment, Intesa Sanpaolo and IBM implemented variational quantum circuit classifiers for payment fraud detection, reporting accuracy improvements over classical classifiers (World Economic Forum, 2025). Rath and Date (2025) proposed a Hybrid Quantum-Classical Deep Neural Network for credit scoring on the UCI German Credit Dataset, matching the performance of XGBoost and classical neural networks on small and medium-sized datasets (Naik et al., 2025). A further practical approach is the QBoost framework (Neven et al., 2012), which formulates feature selection as a QUBO problem solvable on quantum annealers. Pourabdollah et al. (2022) and Acampora et al. (2023) demonstrated that fuzzy inference systems can be mapped onto quantum annealers, offering an interpretable interface for quantum classifiers (Pourabdollah et al., 2022).

Beyond purely numerical financial features, alternative data sources such as news sentiment, social media indicators, and investor attention variables have been shown to carry predictive information for fraud detection and credit risk models (Bollen et al., 2011; Baker and Wurgler, 2006; Hiew et al., 2019). In particular, news sentiment has been found to carry significant predictive information for the cross-section of stock returns in European markets (Gambarelli and Muzzioli, 2025), motivating the systematic incorporation of text-based features alongside numerical financial data in quantum financial classification frameworks.

Despite this progress, the existing quantum fraud detection literature remains confined to single financial sectors. No framework has been tested simultaneously across banking, insurance, FinTech, and microfinance, and no quantum fraud detection approach has been applied specifically to European markets, despite an estimated EUR 4.2 billion in financial fraud across Europe in 2024 (ECB-EBA, 2024) providing a strong practical motivation for such research.

### 2.4 Quantum computing in insurance and actuarial science

The insurance sector shares several structural features with banking, including stochastic claims modelling, risk aggregation under correlation, and large-scale scenario simulation, yet it remains the most underserved application area in quantum finance. This is largely because insurance introduces additional complexities absent from banking, such as multi-year liability structures, mortality dynamics, and Solvency II regulatory capital requirements.

The first systematic study of quantum computational methods in insurance and actuarial science, covering non-life, life, and reinsurance applications, was conducted by Liu et al. (2025). Their results show that quantum stochastic modelling can yield an exponential speedup in memory requirements, quantum optimization can provide a quadratic speedup in computing aggregate loss distributions, and quantum optimization can support the design of reinsurance treaties through coverage-layer selection. Tamturk and Cortis (2023) demonstrated the feasibility of simulating compound Poisson loss processes on IBM Qiskit simulators.

However, all existing quantum insurance studies rely on US or other non-European datasets. The European insurance sector differs substantially in regulatory framework (Solvency II), mortality structure (European mortality tables), and catastrophe risk profile (Alpine floods, Mediterranean droughts, Central European windstorms). Climate risk has become an increasingly important concern for European insurers, affecting both the liability side, through catastrophe claims, and the asset side, through sovereign bond exposure to climate transition risk (Dietz et al., 2016; Capriotti and Muzzioli, 2026).

No existing quantum actuarial study addresses the European insurance context, the Solvency II regulatory framework, or the interaction between climate risk and quantum stochastic insurance modelling. This represents a significant gap given the practical relevance of these issues for European insurers and regulators, and points to a clear opportunity for future research grounded in European data and regulatory requirements. Overall, quantum actuarial science remains one of the least explored application areas, despite its potentially significant practical impact.

### 2.5 Quantum econometrics and mixed-frequency data

The intersection of quantum computing with the econometric tools used daily by financial practitioners represents one of the most novel and underexplored domains in quantum finance research. In an interview conducted by Berkowitz (2025), Ghysels explicitly identifies the translation of established econometric models, including MIDAS, into quantum algorithmic form as a research priority, noting that MIDAS is inherently regression-based and therefore naturally connected to machine learning, making it a suitable candidate for quantum enhancement. This perspective is set out by Ghysels, in an interview with Berkowitz (2025), as a forward-looking research direction rather than an experimental validation. This explicit call to translate established econometric tools into quantum algorithmic form (Berkowitz, 2025) directly motivates the quantum MIDAS research direction developed in Section 3.2.

The MIDAS (Mixed Data Sampling) framework, introduced by Ghysels et al. (2004), addresses the problem of combining financial variables measured at different time frequencies. Daily stock returns, monthly credit spreads, quarterly GDP releases, and annual insurance statistics cannot be meaningfully aggregated without information loss. The standard MIDAS regression is reported in Equation 1:


$$y(t)=\beta_{0}+\beta_{1}\cdot B(L;\theta )\cdot x(t,m)+\varepsilon (t)$$ (1)

where y(t) is a low-frequency outcome, x(t,m) is a high-frequency predictor, B(L;θ) is a polynomial lag-weighting function parameterized by *θ*, and *m* is the frequency ratio. Estimating θ is a constrained nonlinear least squares problem for which quantum acceleration has not previously been investigated.

A natural high-frequency input is volatility indices derived from European option markets, which are known to carry forward-looking information about future realized variance and tail risk at daily frequency (Bakshi et al., 2003; Carr and Wu, 2009); the corresponding low-frequency component is typically provided by macroeconomic data from the ECB Statistical Data Warehouse. News sentiment indicators offer a further high-frequency input with strong empirical support for European markets: Gambarelli and Muzzioli (2025) found that news sentiment carries significant predictive power for the cross-section of European stock returns, with textual and sentiment information adding predictive value beyond standard financial news at the daily frequency. Combining volatility indices and news sentiment as high-frequency components would yield a more empirically grounded and comprehensive mixed-frequency specification than has previously been used in classical MIDAS applications.

Despite the clear theoretical motivation and the widespread use of MIDAS models in financial econometrics, no quantum implementation of MIDAS currently exists anywhere in the literature, representing a clear and well-defined opportunity for future research at the intersection of quantum computing and financial econometrics.

Key takeaways:

- No quantum implementation of MIDAS currently exists anywhere in the literature.
- In an interview with Berkowitz (2025), Ghysels identifies translating econometric tools such as MIDAS into quantum form as a research priority.
- Natural high-frequency inputs: European option-implied moments and news sentiment; low-frequency: ECB macro data.
- Core challenge: estimating the MIDAS lag-weighting function as a constrained nonlinear least-squares/quantum linear-systems problem.

### 2.6 Quantum computing and cryptocurrency security

Quantum computing intersects with cryptocurrency security in three distinct ways: mining, digital signatures, and the broader transition to post-quantum cryptographic standards. The first concerns mining. Bitcoin’s proof-of-work relies on unstructured search, which Grover’s algorithm (Grover, 1996) can in principle solve with a quadratic speedup. In practice, however, this advantage appears economically irrelevant: BTQ Technologies (2025) found that competitive quantum Bitcoin mining at January 2025 difficulty would require approximately 10^23^ physical qubits and 10^25^ watts of energy, comparable to the output of a star, making quantum mining economically and physically infeasible with any foreseeable technology. Nerem and Gaur (2023) similarly showed that the quantum mining advantage collapses once oracle construction overhead and realistic hardware clock rates are accounted for.

The more genuine quantum threat to cryptocurrency concerns digital signatures rather than mining. Bitcoin’s ECDSA (Elliptic Curve Digital Signature Algorithm) and most blockchain signature schemes rely on the elliptic curve discrete logarithm problem, which Shor’s algorithm solves in polynomial time on a fault-tolerant quantum computer. Babbush et al. (2026) estimated that breaking the 256-bit Elliptic Curve Discrete Logarithm Problem (ECDLP-256) would require fewer than 500,000 physical qubits, within the expected 2030–2035 hardware roadmap. More recent estimates point in the same direction: Cain et al. (2026) show that Shor’s algorithm could, in principle, run at cryptographically relevant scales on neutral-atom hardware using as few as 10,000 qubits, and could solve the discrete logarithm on the P-256 curve within days on a system of about 26,000 qubits substantially fewer than earlier estimates. The practical runtime of such qubit-efficient schemes, however, remains debated. Almuhammadi and Alghamdi (2025) reviewed quantum blockchain attack risks and proposed transition protocols for migrating to quantum-resistant architectures. Despite this looming threat, Wu (2025) found that fewer than 0.35% of current blockchain transactions employ post-quantum cryptographic algorithms, indicating an inadequate state of preparation across the industry. The role of sentiment, attention, and uncertainty in cryptocurrency markets (Bernier and Muzzioli, 2026) and the hedging effectiveness of cryptocurrencies in European equity portfolios (Gambarelli et al., 2023; Bouri et al., 2017) further connect this security dimension to mainstream European financial risk management.

In response to these vulnerabilities, NIST finalized its first three post-quantum cryptographic standards in 2024: ML-KEM, ML-DSA, and SLH-DSA (National Institute of Standards and Technology, 2024). Fernandez-Carames and Fraga-Lamas (2020) surveyed the broader landscape of post-quantum distributed ledger technology, while Bonaparte (2025) proposed an integrated quantum blockchain framework. BTQ Technologies (2025) further proposed Proof of Quantum Work (QPoW), estimating a 1,560-fold improvement in energy efficiency over classical proof-of-work. The urgency of this transition is amplified by the growing tokenization of assets by European financial institutions: BlackRock’s BUIDL fund on Ethereum, for example, reached USD 1.5 billion in 2024, with its long-term security depending on a timely post-quantum transition, for which the EU has issued a Coordinated Implementation Roadmap (Commission Recommendation (EU) 2024/1101: European Commission, 2024), while the EU MiCA regulation governs the broader oversight of crypto-assets.

Taken together, these developments suggest that the most pressing quantum security challenge for cryptocurrency markets lies not in mining but in the orderly and timely migration of signature schemes and institutional infrastructure to post-quantum standards, an area where empirical and regulatory research specific to European markets remains limited.

Key takeaways:

- The mining threat (Grover) is economically negligible (≈10^23^ qubits, star-scale energy).
- The real threat is to digital signatures (ECDSA) via Shor; ECDLP-256 needs <500,000 physical qubits (2030–2035 roadmap).
- NIST finalised ML-KEM, ML-DSA and SLH-DSA (2024); fewer than 0.35% of transactions are post-quantum.
- European relevance: institutional asset tokenization (e.g., BlackRock BUIDL) under the EU post-quantum transition roadmap [Commission Recommendation (EU) 2024/1101], with MiCA governing crypto-asset oversight.

### 2.7 Hybrid quantum AI and explainable financial models

The connection between fuzzy logic and quantum computing rests on a solid theoretical foundation within the broader landscape of quantum artificial intelligence (Acampora et al., 2025). Pourabdollah et al. (2022) demonstrated that fuzzy inference engines can be implemented directly on quantum annealers, showing that the selection and firing of fuzzy rules constitutes a natural QUBO problem. This approach was subsequently extended to gate-based quantum computers by Acampora et al. (2023), confirming that fuzzy systems are not only conceptually compatible with quantum hardware but can be practically implemented on it. More recently, Combarro et al. (2023) showed that the Kemeny ranking aggregation problem, a computationally challenging combinatorial optimisation problem in social choice and multi-criteria decision analysis, can be encoded as a QUBO and solved using both quantum annealing and the Quantum Approximate Optimisation Algorithm (QAOA), with their proposed formulation consistently outperforming alternative encodings across the problem instances and hardware configurations tested. These results support the broader thesis that fuzzy-compatible combinatorial problems are natural candidates for quantum optimisation.

The relationship between fuzzy rule-based systems and model explainability in finance is well established in the classical literature. A fuzzy rule base, consisting of interpretable if-then rules such as “IF credit score is low AND debt-to-income ratio is high, THEN default risk is elevated,” is directly readable, auditable, and modifiable by a human expert (Zadeh, 1965; Bellman and Zadeh, 1970; Mendel, 2017). By contrast, decisions produced by quantum circuits, classical neural networks, and gradient boosting models are inherently opaque and not human-readable. The SAFE AI framework (Giudici and Raffinetti, 2023) formalizes this distinction by introducing explainability risk, the lack of clarity, control, and auditability in model outputs, as a measurable risk dimension comparable to market or model risk in financial risk management. Financial models deployed in regulated environments, in particular creditworthiness assessment and pricing in life and health insurance, are subject to mandatory transparency requirements under the EU AI Act (European Parliament, 2024) and the EBA Internal Governance Guidelines (EBA, 2021), making explainability risk a compliance requirement rather than merely a desirable property.

The relationship between fuzzy logic and quantum computing for financial explainability is bidirectional. In one direction, fuzzy logic improves quantum interpretability: fuzzy membership functions convert vague financial inputs, such as analyst ratings, sentiment signals, and ESG assessments, into continuous degrees encodable into quantum states, providing an interpretable front-end for quantum models, while fuzzy defuzzification converts quantum outputs back into human-readable linguistic decisions, providing an interpretable back-end. This is supported by the fuzzy option pricing literature (Muzzioli and De Baets, 2017) and fuzzy regression methodology (Tanaka et al., 1982; Diamond, 1988; Muzzioli et al., 2020), which establish that fuzzy methods are well suited to representing the inherent vagueness of financial information where probabilistic assumptions are overly restrictive.

In the other direction, quantum computing improves fuzzy rule base construction. Identifying the smallest set of fuzzy rules that achieves a desired error level can be formulated as a set-cover problem and mapped onto a QUBO representation (Pourabdollah et al., 2022; Acampora et al., 2023; Neven et al., 2012). Quantum annealing can solve this QUBO more efficiently than classical exhaustive search over large candidate rule sets, yielding more parsimonious and accountable rule bases with substantially lower Explainability risk. This improvement can be evaluated using three measures directly operationalized as part of the SAFE Explainability dimension (Giudici and Raffinetti, 2023; Mendel, 2017): rule count (parsimony), rule complexity (average number of conditions per rule), and coverage (fraction of input space covered). A more recent and methodologically unified approach to this kind of measurement is proposed by Giudici and Kolesnikov (2026), who introduce Rank Graduation Accuracy (RGA), Rank Graduation Robustness (RGR), and Rank Graduation Explainability (RGE), three rank-based metrics derived from a common Lorenz-curve foundation, combined into a single integrated Compliance Score. Their explainability metric, RGE, is conceptually related to the coverage and parsimony measures described above, but is obtained by systematically removing features and measuring the resulting degradation in predictive performance, offering a more rigorous and validated alternative to *ad hoc* rule-counting heuristics.

While the first direction, fuzzy logic enhancing quantum interpretability, has received some theoretical attention, the second direction, quantum optimisation of fuzzy rule bases to reduce financial model Explainability risk, remains virtually absent from the existing literature. This asymmetry represents a clear opportunity for future research at the intersection of fuzzy systems, quantum optimisation, and financial model governance.

Key takeaways:

- Fuzzy inference maps naturally to a QUBO and is implementable on annealers and gate-based hardware.
- The link is bidirectional: fuzzy logic adds interpretability to quantum models; quantum annealing yields more parsimonious rule bases.
- Explainability risk (SAFE AI; Giudici and Raffinetti, 2023) is measurable; RGA/RGR/RGE (Giudici and Kolesnikov, 2026) unify it.
- Under-explored direction: quantum optimisation of fuzzy rule bases to reduce Explainability risk.

### 2.8 Cross-cutting issues: explainability, trustworthy AI, robustness, and governance

The applications reviewed in the preceding sections share a common challenge that cuts across all financial domains: ensuring that quantum-enhanced systems remain safe, explainable, and governable as they move from theoretical proof-of-concept toward practical deployment in regulated financial environments.

A central concern is regulatory compliance. Under the EU AI Act [European Parliament, 2024), credit scoring and creditworthiness assessment (Annex III, point 5(b)] and risk assessment and pricing in life and health insurance (Annex III, point 5(c)) are classified as high-risk AI applications, subject to mandatory requirements for transparency, human oversight, robustness, and audit. Notably, AI systems whose main intended purpose is the detection of financial fraud are expressly excluded from the high-risk credit-scoring category, although they may still fall under other regulatory obligations. New model risks arise specifically when quantum classifiers are used for fraud detection or credit approval if their decision boundaries are not explainable to supervisors, risk managers, and the customers affected. Financial institutions are further bound by the EBA Internal Governance Guidelines (EBA, 2021), which extend these transparency obligations to internal model risk management more broadly, and, in the case of blockchain-based assets, by the EU MiCA regulation for crypto-asset governance and by the EU Coordinated Implementation Roadmap for the transition to post-quantum cryptography [Commission Recommendation (EU) 2024/1101].

These regulatory pressures translate into the SAFE AI framework (Giudici and Raffinetti, 2023), which identifies Security, Accuracy, Fairness, and Explainability as measurable risk dimensions applicable across financial AI systems, quantum or classical alike. The future of quantum ML systems in finance must therefore be judged not only on predictive power, but also on these SAFE AI considerations, including explainability, fairness, resistance to adversarial exploitation, and the reproducibility of model results. Section 2.7 discusses in detail how fuzzy logic offers one concrete mechanism for operationalizing the Explainability dimension of this framework, while more recent work (Giudici and Kolesnikov, 2026) proposes unified, rank-based metrics for measuring Accuracy, Robustness, and Explainability jointly through a single Compliance Score. Recent work by Chen et al. (2026) suggests that hybrid quantum AI models may be more SAFE, and in particular more robust, than classical AI models.

Robustness represents a further cross-cutting concern specific to the quantum setting. Quantum classifiers operating on NISQ hardware are exposed to gate noise and decoherence effects largely absent from classical systems, raising the question of whether a model’s predictions, and its explainability properties, remain stable under realistic hardware imperfections. This robustness dimension has so far received considerably less attention in the literature than explainability, despite being equally relevant to governance and regulatory compliance.

Beyond hardware noise, several fundamental algorithmic limitations constrain quantum financial applications. The data-loading, or input, bottleneck means that encoding classical financial data into quantum states can itself consume resources that offset the theoretical speed-up, a concern that is especially acute for quantum machine learning on large datasets. Hybrid quantum-classical algorithms further incur input/output latency and communication overhead at every iteration of the variational loop, which can dominate total runtime on current cloud-accessed hardware. Because these constraints are intrinsic to the algorithms rather than transient properties of present-day devices, they place a ceiling on the practical advantage achievable in the near term and should be weighed alongside NISQ noise when assessing feasibility.

Taken together, these considerations suggest that the long-term viability of quantum finance applications will depend not only on computational advantage, but on the parallel development of explainable, robust, and well-governed systems capable of meeting the transparency and accountability standards increasingly demanded by European financial regulation.

### 2.9 Research gaps

The review of the literature in the preceding subsections reveals a consistent pattern: rapid theoretical progress in quantum finance has not been matched by empirical validation on European financial data, by coverage of key applied finance sectors beyond banking, or by translation of the most widely used financial econometric tools into quantum form. Five macro-gaps are identified as the primary directions for future research.

#### 2.9.1 Gap 1: Quantum econometric applications

The Quantum Hidden Markov Model framework developed by Ghysels et al. (2025) has not been applied to European financial market data, nor validated using option-implied variance and moment inputs derived from European option markets. Relatedly, a quantum implementation of the Mixed Data Sampling (MIDAS) regression framework remains undeveloped, despite Ghysels, in an interview with Berkowitz (2025), explicitly identifying this translation as a research priority for the field. Both gaps concern the translation of established financial econometric tools, stochastic volatility estimation and mixed-frequency regression, into quantum algorithmic form, and their validation on European data.

#### 2.9.2 Gap 2: Quantum portfolio optimization under realistic constraints

No existing study benchmarks quantum optimisation methods against industry-standard classical solvers (CPLEX, Gurobi) on realistic European portfolio instances incorporating the full simultaneous set of ESG mandate thresholds, Basel IV risk-weighted asset constraints, cardinality bounds, and minimum liquidity requirements. Whether quantum or hybrid methods offer any measurable advantage over best-in-class classical alternatives under these full institutional constraints, using European equity data, remains an open empirical question.

#### 2.9.3 Gap 3: Cross-sector quantum anomaly, default and risk detection

The existing quantum fraud detection literature is confined to single-sector studies. No framework has been developed or validated simultaneously across banking, insurance, FinTech, and microfinance sectors, nor has any quantum fraud detection approach been applied specifically to European markets.

#### 2.9.4 Gap 4: Quantum actuarial science for European insurance

While Liu et al. (2025) published the first systematic quantum insurance study, the European insurance context remains entirely unaddressed: no quantum actuarial research incorporates the Solvency II regulatory framework, European mortality tables, European catastrophe risk profiles, or the interaction between climate risk and insurance liability modelling. The financial applications reviewed in Sections 2.1 to 2.6 share a requirement that becomes binding once these models move toward deployment in regulated markets: their outputs must be explainable, robust, and governable. This motivates the final, cross-cutting macro-gap.

#### 2.9.5 Gap 5: Explainability, robustness, and governance of quantum financial AI

The mutual enhancement between fuzzy logic and quantum computing, whereby fuzzy methods improve the interpretability of quantum models and quantum optimisation improves the parsimony of fuzzy rule bases, has not been formalised as an integrated financial AI framework, nor has it been validated empirically using measurable SAFE Explainability metrics on real financial data. This gap extends beyond explainability alone: no existing study integrates certified robustness against adversarial perturbations and hardware noise, geometric decision-making on Riemannian manifolds, and Bayesian governance of model adoption into a unified framework for trustworthy quantum financial AI. The complementary risks of explainability and robustness have so far been treated in isolation, and no work has translated this integrated agenda into a hardware-aware experimental design executable on currently available quantum computing infrastructure.

## 3 Research directions

Building on the review of the literature and the five macro-gaps identified in Section 2, this section proposes seven directions for future research. These are research questions and proposals, not claimed results. Each direction responds to one or more of the gaps identified above and is grounded in European financial data and currently available quantum computing infrastructure. These seven directions are organised thematically: Sections 3.1–3.2 address econometric applications, Sections 3.3–3.5 address optimisation and risk applications, Section 3.6 addresses explainability, and Section 3.7 introduces a complementary robustness and governance dimension that applies across the preceding six directions.

### 3.1 Quantum stochastic volatility estimation for European markets

A natural first direction concerns the Quantum Hidden Markov Model framework of Ghysels et al. (2025), currently the most testable quantum method for stochastic volatility estimation, which has not yet been applied to European data. A two-state QHMM calibrated on EURO STOXX 600 realized volatility, separating low- and high-volatility regimes, would allow the tighter non-asymptotic bounds derived in that work to be assessed empirically under realistic hardware noise.

Option-implied information offers a natural extension: the VSTOXX and model-free implied moments (Bakshi et al., 2003; Carr and Wu, 2009) carry forward-looking expectations about volatility and tail risk absent from historical variance, with the benchmark of Campisi et al. (2024) and classical filters [Kalman, two-state HMM, GARCH (1,1)] providing the comparison set. A simulator-first validation, followed by hardware tests on reduced instances, is the appropriate empirical path.

### 3.2 Quantum MIDAS and mixed-frequency regression

No quantum implementation of MIDAS exists, despite Ghysels identifying it as a priority in an interview with Berkowitz (2025). A natural target combines daily EURO STOXX 600 returns and VSTOXX implied variance as high-frequency inputs with quarterly ECB indicators (GDP growth, inflation, 3-month Euribor) as low-frequency predictors, yielding a mixed-frequency panel that is both statistically demanding and empirically grounded (Bakshi et al., 2003).

Because the predictors mix continuous and ordinal or categorical variables, where standard regression breaks down, reduced rank regression for mixed variables (De Rooij et al., 2026) offers a way to project the macroeconomic indicators into a low-dimensional latent space before encoding, reducing the linear system size and the burden on the hardware.

The formulation maps the constrained nonlinear least squares estimation of the lag weighting function to a quantum linear systems problem, to be benchmarked against a classical MIDAS baseline on parameter estimates and computational efficiency. The open question is whether quantum linear algebra accelerates convergence or improves estimation quality, and at what problem dimension any advantage first appears.

### 3.3 Quantum portfolio optimization with full European constraints

A further direction applies quantum annealing and variational algorithms to EURO STOXX 600 portfolio optimization with the full simultaneous set of real-world constraints embedded in the QUBO cost Hamiltonian: ESG mandate thresholds, Basel IV risk-weighted asset budgets, quadratic transaction costs, cardinality bounds, and minimum liquidity buffers. The relevant question is whether quantum or hybrid methods gain any advantage over CPLEX and Gurobi on realistic European instances as scale grows from *N* = 20 to *N* = 200 assets.

Dimensionality can be addressed before the quantum loop. Parsimonious time-series clustering via P-splines (Iorio et al., 2016, 2018) smooths return trajectories into functional data, so that a curated subset of cluster-representative assets is passed to the D-Wave or QAOA solver. Robust alternatives to Markowitz (1952), such as the weighted Lp depth function (Pandolfo et al., 2020), handle the heavy tails typical of European equity data and raise the classical benchmark.

Quantum annealing is a natural fit for this problem, since the QUBO with cardinality constraints embeds directly into the annealer’s native topology without circuit compilation overhead; gate-based approaches could be benchmarked in parallel. Either outcome is scientifically relevant: even a finding that classical solvers outperform quantum methods at all tested scales would be a useful and publishable result, clarifying the near-term applicability of quantum portfolio optimisation in practice.

### 3.4 Cross-sector quantum anomaly, default and risk detection for European markets

A multi-sectoral quantum fraud detection framework would allow the leading quantum ML techniques (Quantum Kernel SVM, Quantum Variational Classifiers, QBoost) to be compared against classical baselines (Random Forest, XGBoost, LightGBM) across fraud typologies. Given the structural missingness common in such data, tree-based imputation (D'Ambrosio et al., 2012) keeps the quantum kernels high-fidelity and limits noise propagation on NISQ hardware. Because large labelled European fraud datasets are rarely publicly available, this direction is framed around anomaly, default and risk detection using openly available proxies for method development, with validation on proprietary or partner transaction data envisaged as a subsequent step. Representative public datasets include the UCI “Default of Credit Card Clients” dataset (credit-default classification), EM-DAT European catastrophe records (insurance loss-severity modelling), the aggregated ECB-EBA payment-fraud statistics (used for calibration and benchmarking rather than model training), and the Kiva microfinance database (loan-repayment risk). Genuine cross-sector fraud validation on European transaction-level data remains dependent on access to institutional datasets. This cross-sector design is ideally suited for QBoost (Neven et al., 2012), since its sparse feature selection naturally maps to a QUBO formulation that is well suited to quantum annealing, and can then be compared across sectors. The feature representation is further enhanced by using fuzzy pre-processing of qualitative inputs (Jang, 1993) to transform the financial variables with categorical and ordinal nature into fuzzy membership degrees prior to quantum encoding.

### 3.5 Quantum actuarial science for European insurance under solvency II

A quantum actuarial pipeline under Solvency II points to three tractable, empirically grounded applications. Quantum simulation of aggregate compound Poisson loss distributions on EM-DAT European catastrophe data would deliver the first quantum actuarial results for physical risk events in Europe; a quantum mortality model on Human Mortality Database data for Italy, Germany, France and the United Kingdom would address the longevity-risk side of pension liability management. Third, the combinatorial reinsurance treaty design problem could be formulated as a QUBO and solved through quantum annealing. Integrating climate risk is a further frontier: the interaction of physical risk, carbon-pricing transition risk and sovereign spread dynamics is now central to European Solvency II stress testing (Dietz et al., 2016; Capriotti and Muzzioli, 2026; ECB, 2021), and quantum stochastic models with climate scenario variables would extend the actuarial frontier to the EU Green Deal context.

### 3.6 A bidirectional fuzzy-quantum framework for financial explainability

The most novel direction concerns the bidirectional relationship between fuzzy logic and quantum computing, with model explainability as the central link, and bears on the governance of quantum AI in regulated finance. The SAFE AI framework (Giudici and Raffinetti, 2023) treats Explainability risk as a quantifiable dimension of model risk analogous to market, credit and operational risk; quantum models without an interpretable front-end carry maximum Explainability risk by construction. The open problem is how the bidirectional fuzzy-quantum framework of Section 2.7 can be operationalised and empirically validated.

Explainability is the bridge between fuzzy logic and quantum computing. Fuzzy rule bases provide what quantum models lack: interpretable, auditable, human-readable reasoning. Quantum annealing provides what fuzzy rule base construction lacks: efficient combinatorial optimization producing more parsimonious and more explainable rule sets. The link between these two directions is measurable through three SAFE Explainability indicators: rule count, rule complexity, and coverage.

A first question is whether quantum annealing yields more parsimonious fuzzy rule bases for financial classification than classical algorithms, comparing quantum- and classically-selected rule sets on the SAFE Explainability indicators alongside standard classification metrics. The fuzzy regression methodology of Muzzioli et al. (2020) and the possibilistic portfolio framework of Inuiguchi and Ramik (2000) and Leon et al. (2002) provide the fuzzy calibration methods for the input membership functions.

Global Sensitivity Analysis complements this: it quantifies how sensitive the fuzzy rule bases are to quantum state preparation and hardware noise (Vannucci et al., 2026), while its variable-importance form (Vannucci et al., 2026) filters uninformative features, reducing the qubit count and the initial explainability risk.

For validating quantum fuzzy clustering of EURO STOXX 600 companies against classical fuzzy C-means, the Adjusted Concordance Index (D'Ambrosio et al., 2021), an extension of the Adjusted Rand Index for fuzzy partitions, measures the stability and distinctiveness of the quantum-generated groupings.

A second question is whether quantum fuzzy clustering, mapping Fuzzy C-Means to a QUBO (Bauckhage et al., 2017), produces more interpretable and auditable EURO STOXX 600 groupings than classical clustering. A third, integrative question is whether a quantum-optimised fuzzy rule base that identifies volatility regimes can serve as an interpretable front-end for the QHMM estimator of Ghysels et al. (2025) on EURO STOXX 600 data. Such an integrated fuzzy-quantum volatility pipeline would connect the stochastic volatility and explainability research directions into a single framework: the fuzzy rule base identifies the volatility regime interpretably, the QHMM refines the volatility estimate with quantum-enhanced precision, and SAFE Explainability metrics quantify the transparency of the combined model.

A formal SAFE AI scorecard for quantum financial systems, spanning explainability, robustness to hardware noise, fairness, auditability, cybersecurity resilience and stability across regime shifts, would bridge quantum computational research and financial supervision in the European context.

### 3.7 Robustness certification and geometric decision-making for quantum financial AI

A final, integrative dimension concerns robustness, asking under which conditions the preceding models can serve as decision-support systems in regulated institutions. Explainability alone is insufficient: an interpretable quantum classifier may still be unstable to input perturbations, NISQ gate noise, or regime switches, so each model should carry a certified neighbourhood within which its decision is provably invariant. Three lines compose this direction. The first is geometric: admissible portfolio weights under simultaneous ESG, Basel IV and cardinality constraints form not a Euclidean simplex but a stratified manifold, and regime-switching equilibrium under ambiguity is naturally a Nash equilibrium on a Riemannian manifold (Ghysels and Morgan, 2024). The differential-geometric optimisation programme of the Udriste school provides the infrastructure to translate these problems onto such manifolds, turning the QUBO cost Hamiltonian into a curvature-aware penalty. Empirical implementation should test whether quantum annealing with geometry-aware penalty calibration produces solutions closer to the constrained-manifold optimum than naive Euclidean QUBO formulations on the EURO STOXX 600 universe.

The second line concerns certified robustness under adversarial and hardware perturbations. Quantum classifiers deployed in credit scoring, payment fraud detection and insurance pricing operate on input vectors that include sentiment indicators, ESG scores and behavioural features which can be adversarially manipulated by counterparties with non-trivial incentives. The classical robustness certification literature, based on Lipschitz bounds and randomised smoothing, has no direct quantum counterpart, and the interaction between adversarial input perturbations and the intrinsic gate noise of NISQ hardware is largely unexplored. A hardware-aware certification protocol would annotate a quantum classifier with two radii: an input-side radius bounding the perturbation under which the decision is preserved with prescribed probability, and a circuit-side radius bounding the tolerable gate noise on the target device. Gate-based hardware integrated with classical supercomputing offers a natural setting for this protocol.

The third line concerns the governance of quantum AI adoption in financial institutions. The decision to deploy a quantum model in production is itself a sequential decision under uncertainty in which the model evidence accumulates across simulator validation, hardware validation and shadow deployment, and the regulatory cost of premature adoption interacts non-trivially with the strategic cost of late adoption relative to competitors. A dynamic Bayesian governance framework, extending the model adoption literature to quantum financial AI, provides the formal instrument to manage this trade-off, with posterior beliefs over model reliability updated at each validation stage and the deployment decision triggered when the certified robustness radii cross sector-specific regulatory thresholds. This third line connects naturally to the EBA Internal Governance Guidelines (EBA, 2021) and EU AI Act (European Parliament, 2024) compliance requirements, and closes the loop between technical robustness certification and the institutional decision to bring a quantum financial model into operation.

Together, the three lines form an evaluation design cutting across the previous six directions: each model of Sections 3.1 to 3.6 would be assessed against the same geometric, robustness and governance criteria, yielding a SAFE-and-ROBUST scorecard rather than a single accuracy figure, again on a simulator-first basis. Rather than a new algorithm, this direction proposes a methodological standard for evaluating quantum financial models.

## 4 Conclusion

Quantum computing is emerging as a promising computational paradigm for finance. Although current applications remain at an early stage, the literature demonstrates growing interest across optimization, pricing, machine learning, econometrics, and risk management. The field remains characterized by limited empirical validation and significant hardware constraints, but rapid advances in quantum infrastructure and hybrid quantum-classical methods suggest substantial opportunities for future research. The growing availability of European quantum computing infrastructure may play an important role in the next phase of quantum finance research and experimentation.

This paper has reviewed the current state of quantum computing in finance across seven domains: portfolio optimization, derivative pricing and stochastic volatility, machine learning for fraud detection and credit risk, insurance and actuarial science, mixed-frequency econometrics, fuzzy-quantum hybrid methods, and cryptocurrency security, and identified seven directions for future research that are, to the best of our knowledge, absent from the existing international literature.

The seven research directions share three structural characteristics that distinguish them from much of the existing quantum finance literature. First, they are all grounded in European financial data (e.g., EURO STOXX 600 equities, VSTOXX option-implied volatility, EU ETS carbon markets, European mortality tables, and EM-DAT European catastrophe data) addressing the limited coverage of European markets in the existing quantum finance literature. Second, they are all designed to be practically executable on accessible quantum computing infrastructure rather than depending on expensive proprietary cloud access to US quantum hardware. Third, they are all structured around a simulator-first, hardware-second validation protocol that ensures publishable scientific results at each stage, regardless of whether the hardware experiments confirm or qualify the simulator findings.

The most scientifically distinctive contribution of this review is the bidirectional fuzzy-quantum explainability framework proposed in Section 3.6. To the best of our knowledge, the relationship between quantum optimization of fuzzy rule bases and measurable reduction in Explainability risk, as defined within the SAFE AI framework, has not been previously established in the literature. This direction translates a methodological observation about fuzzy rule base optimization into a quantifiable, empirically testable, and regulatorily grounded contribution to responsible financial AI. The EU AI Act (European Parliament, 2024) imposes explainability requirements for high-risk AI applications, including credit scoring, insurance pricing, and financial market management, making the development of quantum methods that can measurably enhance model interpretability and auditability a practical regulatory necessity rather than an academic aspiration.

The case for sustained investment in quantum finance research, spanning algorithm design, empirical validation, and infrastructure development, is particularly compelling at the current stage of hardware development, as argued by Ghysels in an interview with Berkowitz (2025). The growing availability of national and European quantum computing infrastructure creates favourable conditions for research institutions to engage with this frontier, and the directions proposed in this paper are intended to provide a concrete and empirically grounded starting point for that engagement.

Ultimately, the future of quantum finance will depend not only on advances in quantum hardware but also on the development of reliable, interpretable, and empirically validated AI systems capable of supporting financial decision making in regulated environments. It is at this intersection of computational advantage and trustworthy financial AI that the most significant scientific and practical opportunities for European research are likely to emerge.
